# Serial dictatorship mechanisms with reservation prices: heterogeneous objects

**DOI:** 10.1007/s00355-020-01303-w

**Published:** 2021-02-13

**Authors:** Bettina Klaus, Alexandru Nichifor

**Affiliations:** 1 Faculty of Business and Economics, Internef, University of Lausanne, 1015 Lausanne, Switzerland; 2 Faculty of Business and Economics, University of Melbourne, 111 Barry Street, Parkville, VIC 3010 Australia

## Abstract

We adapt a set of mechanisms introduced by Klaus and Nichifor (Econ Theory 70:665–684, 2020), *serial dictatorship mechanisms with (individual) reservation prices*, to the allocation of heterogeneous indivisible objects, e.g., specialist clinic appointments. We show how the characterization of serial dictatorship mechanisms with reservation prices for homogeneous indivisible objects (Klaus and Nichifor 2020, Theorem 1) can be adapted to the allocation of heterogeneous indivisible objects by adding *neutrality*: mechanism $\varphi$ satisfies *minimal tradability*, *individual rationality*, *strategy-proofness*, *consistency*, *independence of unallocated objects*, *neutrality*, and *non wasteful tie-breaking* if and only if there exists a reservation price vector *r* and a priority ordering $\succ$ such that $\varphi$ is a *serial dictatorship mechanism with reservation prices* based on *r* and $\succ$.

## Introduction

We consider the problem of allocating heterogeneous indivisible objects to agents when each agent receives at most one object and pays a non-negative price. Each agent’s preferences over receiving an object and his own payment are given by a general utility function that is not necessarily quasilinear. A *mechanism* selects an *outcome* for the problem by allocating an object and specifying a payment for each agent, i.e., it selects an *allotment* for each agent. We are interested in mechanisms that have desirable properties. More specifically, we consider mechanisms that: satisfy mild efficiency criteria (*minimal tradability* and *non-wasteful tie-breaking*); induce voluntarily participation (*individual rationality*); elicit agents’ true preferences over objects (*strategy-proofness*); select outcomes that are robust, in the sense of remaining invariant when agents leave from the problem with their allotments (*consistency*) or when we remove undesired objects (*independence of unallocated objects*); and in which the names of the objects do not matter and the objects can be relabelled while the payments are kept invariant (*neutrality*).

We show that a mechanism $\varphi$ satisfies all the properties mentioned above if and only if there exists a reservation price vector *r*, which specifies an individual reservation price for each agent that is the same for all objects, and a priority ordering $\succ$ over the set of agents, such that $\varphi$ is a *serial dictatorship mechanism with reservation prices* that is based on *r* and $\succ$ (Theorem 1). Our characterization is tight in the sense that each property used is indispensable.

Intuitively, a serial dictatorship mechanism with reservation prices works as follows. Agents sequentially get to choose feasible objects according to their priority, where the feasible objects are those remaining after all preceding agents made their choices: If the choosing agent’s value for his most preferred object among the feasible ones exceeds his reservation price, he takes the object and he pays his reservation price; otherwise, he receives and pays nothing.

Our model, properties, and mechanisms, are well-suited for understanding markets in which: wealth inequality among agents can be substantial; income redistribution is not feasible; sequential priorities as a main criteria for rationing demand are considered *fair* (Konow 2003) and *just* (Dold and Khadjavi 2017)—and are thus desirable, while maintaining compatibility with some payments is also required. An example of such markets, albeit in a less general model than ours, is the allocation of similar “consultant-led” medical procedures in Australia (Klaus and Nichifor 2020, Section 5); we briefly revisit this example in Sect. 4, after we present our main result (Theorem 1).

Our work is closely related to that of Klaus and Nichifor (2020). We extend their homogeneous indivisible objects model, normative properties, and the class of serial dictatorship mechanisms with reservation prices that they introduced, to heterogeneous indivisible objects. Our characterization (Theorem 1), which uses one additional key property, *neutrality*, provides a counterpart to the main result of Klaus and Nichifor (2020, Theorem 1).^1^

For settings in which agents’ preferences are given by linear orders and there are no payments, several characterizations of the classical serial dictatorship mechanisms are available, e.g., Svensson (1994, 1999), Ergin (2000), and Ehlers and Klaus (2007). Relative to those settings, we extend the model to allow for general utility functions, and we adapt the properties used to characterize classical serial dictatorship mechanisms, as well as the mechanism itself, in a way in which we maintain sequential priorities as the main rationing criteria (as desired), but we relax the earlier limitations that ruled out any payments. Our model and key properties end up being closer to those of Tadenuma and Thomson (1991) and Svensson and Larsson (2002), who do not characterize any mechanism as such;^2^ in contrast, we characterize the class of *serial dictatorship mechanisms with reservation prices*. Furthermore, in our characterization, the reservation price vector and the priority ordering are both derived from the properties, together with the serial dictatorship mechanism with reservation prices that is based on them; in this sense, our approach is related to the recent characterizations of deferred acceptance mechanisms (Kojima and Manea 2010; Ehlers and Klaus 2014, 2016) in which the priorities (or more generally, the choice functions) that the mechanisms are based on, and the mechanisms, are all derived together from the properties.

## Model

Our model extends the homogeneous objects model of Klaus and Nichifor (2020) to heterogeneous objects; to ease the comparison, our exposition and notation are closely based on that of Klaus and Nichifor (2020).

A set of heterogeneous indivisible objects are to be allocated to a set of agents; the sets of objects and the set of agents can change. Let ${\mathbb {N}}$ be the set of potential agents and ${\mathcal {N}}$ be the set of all non-empty finite subsets of ${\mathbb {N}}$, ${\mathcal {N}} \equiv \left\{ N \subseteq {\mathbb {N}} : 0< |N| < \infty \right\}$. Let ${\mathbb {O}}$ be the set of potential real objects and ${\mathcal {O}}$ be the set of all non-empty finite subsets of ${\mathbb {O}}$, ${\mathcal {O}} \equiv \left\{ O \subseteq {\mathbb {O}} : 0< |O| < \infty \right\}$. We assume that $|{\mathbb {O}}|>1$ and that ${\mathbb {O}}$ is infinite.^3^ By 0 we denote the *null object*, which represents not receiving a real object in ${\mathbb {O}}$.

For any set of agents $N\in {\mathcal {N}}$ and any set of real objects $O\in {\mathcal {O}}$, an *allocation vector* $a=(a_{i})_{i\in N}\in \big (O\cup \{0\}\big )^{N}$ such that [for any two agents $i,j\in N$, $i\ne j$, $a_i=a_j$ implies $a_i=a_j=0$] describes which agent in *N* receives which object in $O\cup \{0\}$; we allow for the possibilities that no real objects, or only some, are allocated. We denote the *set of allocation vectors* for a set of agents $N\in {\mathcal {N}}$ and a set of real objects $O\in {\mathcal {O}}$ by ${\mathcal {A}}(N,O).$

We assume that an agent $i\in {\mathbb {N}}$ pays a non-negative *price* $p_i\in {\mathbb {R}}_+$, and we denote the *set of payment vectors* for a set of agents $N\in {\mathcal {N}}$ by ${\mathcal {P}}(N) \equiv \left\{ p=(p_i)_{i\in N}: p \in {\mathbb {R}}^{N}_+\right\}$.

We assume that agents only care about the object they receive and their own payment. Each agent $i\in {\mathbb {N}}$ has preferences that are: (i) for any object, strictly decreasing in the price paid; (ii) such that for any real object, either there exists a price which makes the agent indifferent between [receiving the real object at this price] and [receiving the null object and paying nothing], or he strictly prefers to [obtain the real object, whatever the price] over [receiving the null object and paying nothing], or he strictly prefers to [receive the null object and pay nothing] over [obtaining the real object, whatever the price]; and (iii) strict when comparing any two real objects at the same price. Formally, for each $O \in {\mathcal {O}}$ and each $i\in {\mathbb {N}}$, we represent agent *i*’s preferences by a utility function $u_i:(O\cup \{0\})\times {\mathbb {R}}_+\rightarrow {\mathbb {R}}$ that satisfies the following three properties:

(i) if $0 \le p'_i < p_i$, then for each $o\in O$, $u_i(o,p'_i) > u_i(o,p_i)$ and $u_i(0,p'_i) > u_i(0,p_i)$;
(ii) for each $o\in O$, either
  [there exists a price $v_{i,o}$ such that $u_i(o,v_{i,o})=u_i(0,0)$], or
  [for each $p_i\ge 0$, we have $u_i(o,p_i)>u_i(0,0)$ and $v_{i,o}\equiv \infty$] or
  [for each $p_i\ge 0$, we have $u_i(0,0)>u_i(o,p_i)$ and $v_{i,o}\equiv -\infty$];
  $v_i=(v_{i,o})_{o\in O}$ is agent *i*’s *valuation vector*^4^ and we set $v_{i,0}=0$.
(iii) Agent *i*’s preferences over real objects are strict in the sense that for any pair of real objects $o_1,o_2\in O$, $o_1\ne o_2$, and any payment $p_i$, we have $u_i(o_1,p_i)\ne u_i(o_2,p_i)$;
  hence, for each set of real objects $O\in {\mathcal {O}}$ and any payment $p_i$, the best real object for agent *i* in *O* is well defined and we denote it by $\mathrm {top}_i(O,p_i) = \arg \max _{o \in O}\{u_i(o,p_i)\}$.

Given $u_i:(O\cup \{0\})\times {\mathbb {R}}_+\rightarrow {\mathbb {R}}$ and $o\in O$, $u_{i,o}:{\mathbb {R}}_+\rightarrow {\mathbb {R}}$ denotes agent *i*’s *induced utility function for object o*, i.e., for any payment $p_i$, $u_{i,o}(p_i)=u_i(o,p_i)$.

An example of an agent *i*’s preferences are *quasilinear preferences* $u_i$ with valuation vector $v_i$ such that for all $o_1,o_2\in O$, $o_1\ne o_2$, $v_{i,o_1}\ne v_{i,o_2}$ and for each $(a_i,p_i)\in (O\cup \{0\})\times {\mathbb {R}}_+$, $u_i(a_i,p_i)= v_{i,a_i} - p_i$.

For any set of agents $N \in {\mathcal {N}}$ and any set of real objects $O \in {\mathcal {O}}$, we denote the *set of utility (function) profiles* by ${\mathcal {U}}(N,O)$ and the associated *set of valuation (vector) profiles* by ${\mathcal {V}}(N,O)$.

A *problem* $\gamma$ is specified by a triple (*N*, *O*, *u*) such that $(N,O) \in {\mathcal {N}} \times {\mathcal {O}}$ and $u\in {\mathcal {U}}(N,O)$. We denote the *set of all problems* for $(N,O) \in {\mathcal {N}} \times {\mathcal {O}}$ by $\Gamma (N,O)$.

An *outcome* for any problem $\gamma \in \Gamma (N,O)$ consists of an allocation vector $a\in {\mathcal {A}}(N,O)$ and a payment vector $p\in {\mathcal {P}}(N)$. We denote the *set of outcomes* for a problem $\gamma \in \Gamma (N,O)$ by ${\mathcal {O}} (N,O)\equiv {\mathcal {A}}(N,O) \times {\mathcal {P}}(N).$

A *mechanism* $\varphi$ is a function that assigns an outcome to each problem. Formally, for each $(N,O) \in {\mathcal {N}}\times {\mathcal {O}}$ and each $\gamma \in \Gamma (N,O)$, $\varphi (\gamma ) \in {\mathcal {O}}(N,O)$. Note that we can also represent a mechanism $\varphi$ by its *allocation rule* $\alpha$ and *payment rule* $\pi$, i.e., for each $(N,O) \in {\mathcal {N}}\times {\mathcal {O}}$ and each $\gamma \in \Gamma (N,O)$, $\alpha : \Gamma (N,O) \rightarrow {\mathcal {A}}(N,O)$, $\pi : \Gamma (N,O) \rightarrow {\mathcal {P}}(N)$, and $\varphi (\gamma )=(\alpha (\gamma ),\pi (\gamma ))$. We denote the *allotment* of agent *i* at outcome $\varphi (\gamma )$ by $\varphi _i(\gamma ) = (\alpha _i(\gamma ),\pi _i(\gamma ))$.

Given $N\in {\mathcal {N}}$, a vector $x\in {\mathbb {R}}^N$, and $M\subseteq N$, let $x_{M} \equiv (x_{i})_{i\in M}\in {\mathbb {R}}^M$ be the restriction of vector *x* to the subset of agents *M*. We also use the notation $x_{-i}=x_{N\backslash \{i\}}$. For example, $({\bar{x}}_{i},x_{-i})$ denotes the vector obtained from *x* by replacing $x_{i}$ with ${\bar{x}}_{i}$. We use corresponding notational conventions for utility profiles.

### Properties of mechanisms

Our first property ensures that if there are at least as many agents as objects, then there is some utility profile at which all objects are allocated.

#### Definition 1

(*Minimal Tradability*) A mechanism $\varphi$ satisfies *minimal tradability* if for each $(N,O) \in {\mathcal {N}}\times {\mathcal {O}}$ such that $|O|\le |N|$, there exists a utility profile $u\in {\mathcal {U}}(N,O)$ such that $\bigcup _{i\in N}\{\alpha _i(N,O,u)\}=O$.

*Minimal tradability* was first introduced for single-object problems by Sakai (2013), and then extended to problems with homogeneous objects by Klaus and Nichifor (2020). Our definition of *minimal tradability* coincides with that of Sakai’s (2013) for single-object problems, but it is in character less demanding than that of Klaus and Nichifor (2020).^5^

For $(N,O) \in {\mathcal {N}}\times {\mathcal {O}}$, an outcome $(a,p)\in {\mathcal {O}}(N,O)$ is *individually rational* for utility profile $u\in {\mathcal {U}}(N,O)$ with associated valuation vector $v\in {\mathcal {V}}(N,O)$ if for each $i\in N$, we have $u_i(a_i,p_i)\ge u_i(0,0)$, or equivalently,

1. [$$a_i=0$$ implies $$p_i= 0$$] and
2. [for each $o\in O$, $a_i=o$ implies $p_i\le v_{i,o}$].

By requiring a mechanism to only choose individually rational outcomes, we express the idea of voluntary participation.

#### Definition 2

(*Individual Rationality*) A mechanism $\varphi$ satisfies *individual rationality* if for each $(N,O) \in {\mathcal {N}}\times {\mathcal {O}}$ and each $\gamma \in \Gamma (N,O)$, $\varphi (\gamma )$ is an *individually rational* outcome.

*Strategy-proofness* requires that no agent can benefit from misrepresenting his preferences.

That is, a mechanism is *strategy-proof* if (in the associated direct revelation game) it is a weakly dominant strategy for each agent to report his utility truthfully.

#### Definition 3

(*Strategy-Proofness*) A mechanism $\varphi$ satisfies *strategy-proofness* if for each $(N,O) \in {\mathcal {N}}\times {\mathcal {O}}$, each $(N,O,u)\in \Gamma (N,O)$, each $i \in N$, and each $u'_i$ such that $u'\equiv (u'_i,u_{-i})\in {\mathcal {U}}(N,O)$, $u_i(\varphi _i(N,O,u)) \ge u_i(\varphi _i(N,O,u'))$.

To introduce our next property, *consistency*, which is a key requirement in many frameworks with variable populations, we first define what a *reduced problem* is.

Let $(N,O)\in {\mathcal {N}}\times {\mathcal {O}}$, $\gamma =(N,O,u)\in \Gamma (N,O)$, and $M\subseteq N$. When the set of agents *M* leaves problem $\gamma$ with their $\bigcup _{i\in M}\alpha _i(\gamma )$ allocated objects, the set of remaining objects is $O_{N{\setminus } M}=O{\setminus } \bigcup _{i\in M}\alpha _i(\gamma )$. Hence, the *reduced problem* is $\gamma _{N{\setminus } M}=(N{\setminus } M,O_{N{\setminus } M},u_{N{\setminus } M})$, where $u_{N{\setminus } M}\in {\mathcal {U}}(N{\setminus } M,O_{N{\setminus } M})$ is obtained from *u* by deleting the utilities of agents in *M*, as well as deleting the remaining $N {\setminus } M$ agents’ utilities for the objects $\bigcup _{i\in M}\alpha _i(\gamma )$ that were removed.^6^

*Consistency* is an invariance requirement of the solution if some agents leave together with their allotments. That is, *consistency* requires that if some agents leave with their allotments, then the allocation and the payment for all remaining agents should not change in the resulting reduced problem.

#### Definition 4

(*Consistency*) A mechanism $\varphi$ satisfies *consistency* if for each $(N,O)\in {\mathcal {N}}\times {\mathcal {O}}$, each $\gamma \in \Gamma (N,O)$, and each $M\subseteq N$, we have $\varphi (\gamma _{N{\setminus } M})=\varphi (\gamma )_{N{\setminus } M}$.

*Consistency*, first introduced by Thomson (1983), is one of the key properties in many frameworks with variable populations (see Thomson 2015). We use a similar notion of *consistency* as Tadenuma and Thomson (1991) do (since our models are similar) but adapt it to apply to functions (they allow for correspondences), and we decompose it into two properties: our *consistency* together with our next property, *independence of unallocated objects*, corresponds to the direct adaptation of Tadenuma and Thomson’s *consistency* to our model.

Next, we require that if not all objects are allocated, removing some of the unallocated objects leaves the outcome unchanged.

#### Definition 5

(*Independence of Unallocated Objects*) A mechanism $\varphi$ satisfies *independence of unallocated objects* if for each $(N,O) \in {\mathcal {N}}\times {\mathcal {O}}$, each $(N,O,u) \in \Gamma (N,O)$, and each $O'\subseteq O{\setminus } \bigcup _{i\in N}\alpha _i(N,O,u)$, we have $\varphi (N,O,u)=\varphi (N,O{\setminus } O',u_{O{\setminus } O'})$, where $u_{O{\setminus } O'}\in {\mathcal {U}}(N,O{\setminus } O')$ is obtained from *u* by deleting the utility information for removed objects.

*Independence of unallocated objects* was introduced for a homogeneous objects allocation model by Klaus and Nichifor (2020) who required that removing all unallocated objects leaves an outcome unchanged. We extend their property to allow for heterogeneous objects, and we require that only *some*, but not necessary *all*, unallocated objects be removed.

To introduce our next property, *neutrality*, which is a key requirement in many frameworks in which the names of the objects should not matter in the allocation process, we first define what a *relabelling of the objects* is.

For each $O\in {\mathcal {O}}$, a *relabelling of the objects* is given by a permutation function $\sigma : O \cup \{0\} \rightarrow O \cup \{0\}$ with $\sigma (0)=0$, i.e., under $\sigma$ the names of the real objects are exchanged, e.g., object $o \in O$ becomes object $\sigma (o) \in O$, while the naming of the null object remains unchanged. We denote the *set of relabellings* for a set of real objects $O\in {\mathcal {O}}$ by ${\mathcal {S}}(O)$.

Let $(N,O) \in {\mathcal {N}}\times {\mathcal {O}}$ and $\sigma \in {\mathcal {S}}(O)$. Then, for each utility profile $u\in {\mathcal {U}}(N,O)$ with associated valuation vector $v\in {\mathcal {V}}(N,O)$, a *relabelling of the utility profile* $u^{\sigma }\in {\mathcal {U}}(N,O)$ with associated *relabelling of valuation vector profile* $v^{\sigma }\in {\mathcal {V}}(N,O)$ is such that for each $i \in N$ and each $o \in O$, we have $u_{i,o}^{\sigma }=u_{i,\sigma ^{-1}(o)}$ and $v_{i,o}^{\sigma }=v_{i,\sigma ^{-1}(o)}$.

For example, consider $N=\{1,2,3\}$, $O=\{a,b,c\}$, utility profile $u\in {\mathcal {U}}(N,O)$ with associated valuation vector $v\in {\mathcal {U}}(N,O)$, and a relabelling $\sigma (a)=b$, $\sigma (b)=c$, $\sigma (c)=a$, and $\sigma (0)=0$. Then, for each agent $i \in N$, $u_i=(u_{i,a},u_{i,b}, u_{i,c})$ and $v_i=(v_{i,a},v_{i,b}, v_{i,c})$ are relabelled by $\sigma$ to $u_i^{\sigma }=(u^{\sigma }_{i,a},u^{\sigma }_{i,b}, u^{\sigma }_{i,c})=(u_{i,c},u_{i,a}, u_{i,b})$ and $v_i^{\sigma }=(v^{\sigma }_{i,a},v^{\sigma }_{i,b}, v^{\sigma }_{i,c})=(v_{i,c},v_{i,a}, v_{i,b})$.

A mechanism is *neutral* if a *relabelling of the objects* results in each agent being allocated the object that is the relabelled version of the object that he was previously allocated, while the payments for all agents remain the same as before.

#### Definition 6

(*Neutrality*) A mechanism $\varphi$ satisfies *neutrality* if for each $(N,O) \in {\mathcal {N}}\times {\mathcal {O}}$, each $\sigma \in {\mathcal {S}}(O)$, and for each $i \in N$, we have

$$\begin{aligned} \alpha _i(N,O,u^{\sigma })=\sigma (\alpha _i(N,O,u)) \text{ and } \pi _i(N,O,u^{\sigma })=\pi _i(N,O,u). \end{aligned}$$

For our three agent and three object example above, if say $\alpha (N,O,u)=(a,b,c)$ and $\pi (N,O,u)=(5,0,1)$, then *neutrality* would imply that $\alpha (N,O,u^{\sigma })=(b,c,a)$ and $\pi (N,O,u^{\sigma })=(5,0,1)$.

*Neutrality* was first introduced by Smith (1973) in a voting context. We use the same notion of *neutrality* as Svensson and Larsson (2002) do, and our models are similar, except that we allow for more general preferences than Svensson and Larsson who require quasilinear preferences.

Our last property requires that a mechanism does not select an outcome where an agent is indifferent between [receiving a real object at some price] and [not receiving an object and not paying anything, i.e., withdrawing from the market].

#### Definition 7

(*Non Wasteful Tie-Breaking*) A mechanism $\varphi$ satisfies *non wasteful tie-breaking* if for each $(N,O) \in {\mathcal {N}}\times {\mathcal {O}}$, each $\gamma \in \Gamma (N,O)$, each $i\in N$, and each $o\in O$, $\alpha _i(\gamma )=o$ implies that $u_i(o,\pi _i(\gamma )) \ne u_i(0,0)$.

*Non wasteful tie-breaking*, first introduced by Klaus and Nichifor (2020), is a mild *efficiency* requirement: The *tie-breaking*, which rules out allocating a real object at some price to an agent who is indifferent between such an allotment and withdrawing from the market, is *non-wasteful* in that it keeps the object available, because another agent might strictly prefer to receive it.

## Serial dictatorships with reservation prices

We adapt the class of *serial dictatorships with reservation prices* introduced by Klaus and Nichifor (2020) for the allocation of *homogeneous* indivisible objects to our model with *heterogeneous* indivisible objects as follows.

First, we need to define and fix *reservation prices* and a *priority ordering*.

We assume that for each agent $i\in {\mathbb {N}}$ a *(fixed) reservation price* $f_i\ge 0$ exists. We interpret $f_i$ as the price at which a real object can be allocated to agent *i*. Note that the reservation price $f_i$ is the same for different real objects. We denote a *vector of (fixed) reservation prices* for the set of potential agents ${\mathbb {N}}$ by $f=(f_i)_{i\in {\mathbb {N}}}$ and by ${\mathcal {F}}$ we denote the *set of all (fixed) reservation price vectors* for ${\mathbb {N}}$.

A *priority ordering* $\vartriangleright$ over the set of potential agents ${\mathbb {N}}$ is a complete, asymmetric, and transitive binary relation, with the interpretation that for any two distinct agents $i,j\in {\mathbb {N}}$, $i\vartriangleright j$ means that *i* has a higher priority than *j*. Note that the priority ordering $\vartriangleright$ is the same for different real objects. Let ${\mathcal {P}}$ denote the *set of all priority orderings* over ${\mathbb {N}}$.

Given a reservation price vector $f\in {\mathcal {F}}$ and a priority ordering $\vartriangleright \in {\mathcal {P}}$, the *serial dictatorship mechanism with reservation prices* based on *f* and $\vartriangleright$ is denoted by $\psi ^{(f,\vartriangleright )}$ and determines an outcome for each problem $\gamma = (N,O,u)\in \Gamma (N,O)$ with associated valuation vector $v\in {\mathcal {V}}(N,O)$ as follows.

**Step 0:** If there are no real objects to allocate, then stop and all agents receive and pay nothing. Otherwise, continue.

**Step 1:** The agent with the highest priority in *N* is considered. Let $i\in N$ be this agent.

- If for his most preferred object $\mathrm {top}_i(O,f_i)$ in *O*, $v_{i,\mathrm {top}_i(O,f_i)}> f_i$, then agent *i* obtains $\mathrm {top}_i(O,f_i)$ and pays $f_i$. Set $O_1:=O{\setminus } \{\mathrm {top}_i(O,f_i)\}$. If $O_1=\emptyset$, then we stop and all remaining agents receive and pay nothing. Otherwise, continue.
- If $v_{i,\mathrm {top}_i(O,f_i)}\le f_i$, then agent *i* receives and pays nothing. Set $O_1:=O$ and continue.

**Step** ***l***: The agent with the $l^{\mathrm {th}}$ highest priority in *N* is considered. Let $j\in N$ be this agent.

- If for his most preferred object $\mathrm {top}_j(O_{l-1},f_j)$ in $O_{l-1}$, $v_{j,\mathrm {top}_j(O_{l-1},f_j)}> f_j$, then agent *j* obtains $\mathrm {top}_j(O_{l-1},f_j)$ and pays $f_j$. Set $O_l:=O_{l-1}{\setminus } \{\mathrm {top}_j(O_{l-1},f_j)\}$. If $O_l=\emptyset$, then we stop and all remaining agents receive and pay nothing. Otherwise, continue.
- If $v_{j,\mathrm {top}_j(O_{l-1},f_j)}\le f_j$, then agent *j* receives and pays nothing. Set $O_l:=O_{l-1}$ and continue.

We continue until either all real objects are allocated or all agents have been considered. We denote the resulting outcome by $\psi ^{(f,\vartriangleright )}(N,O,u)$.^7^

## Characterization

### Theorem 1

*A mechanism* $\varphi$ *satisfies* *minimal tradability*, *individual rationality*, *strategy-proofness*, *consistency*, *independence of unallocated objects*, *neutrality*, *and* *non wasteful tie-breaking* *if and only if there exist a reservation price vector* $r\in {{\mathcal {F}}}$ *and a priority ordering* $\succ \in {\mathcal {P}}$ *such that* $\varphi$ *is a serial dictatorship mechanism with reservation prices based on* *r* *and* $\succ$, i.e., $\varphi =\psi ^{(r,\succ )}$.

We formally prove Theorem 1 in Appendix A. Below, we discuss how our result relates to that of Klaus and Nichifor (2020, Theorem 1), and we explain and sketch the more involved uniqueness part of our proof.

Recall that we extended the homogeneous indivisible objects model of Klaus and Nichifor (2020), their normative properties, and their class of serial dictatorship mechanisms with reservation prices to heterogeneous indivisible objects. For our characterization (Theorem 1), we use all the properties that are used by Klaus and Nichifor (2020, Theorem 1), suitably adapted from homogeneous to heterogeneous objects, to which we add one new key property: *neutrality*.

Except for *neutrality*, Klaus and Nichifor (2020, Section 5) interpret properties and mechanisms similar to those in our Theorem 1 in the context of allocating similar “consultant-led” medical procedures (e.g., mole removal surgeries) to patients in Australia. The same intuition and interpretation can be used for the allocation of heterogeneous objects by assuming that these objects are composed of a homogeneous item, corresponding to a specific medical procedure, together with a unique time slot.^8^ Furthermore, for such heterogeneous objects, *neutrality* has the natural interpretation that it requires the out-of-pocket cost paid by a patient for the underlying medical procedure to be the same, regardless of when this procedure is scheduled.^9^

In our characterization (Theorem 1), as well as in that of Klaus and Nichifor (2020, Theorem 1), the uniqueness proof consists of four parts; next, we sketch these parts, highlighting the role played by *neutrality*.

### Proof Sketch

 (Uniqueness) We assume that $\varphi$ satisfies all the properties in the theorem, and then proceed as follows:

1. we construct the individual reservation price vector $r\in {\mathcal {F}}$ (*neutrality* here implies that each agent’s reservation price is the same for any real object);
2. we construct the priority ordering $\succ \in {\mathcal {P}}$ over ${\mathbb {N}}$ (*neutrality* here implies that the priority ordering is the same for any real object);
3. for single-object problems, by Klaus and Nichifor (2020, Proof of Theorem 1, Part 3) it follows that $\varphi =\psi ^{(r,\succ )}$; and
4. we extend the single-object result that $\varphi =\psi ^{(r,\succ )}$ to any set of real objects $O\in {\mathcal {O}}$ via an induction argument.

Parts 1 and 2 bear some resemblance to the corresponding proofs of the main result in Klaus and Nichifor (2020, Proof of Theorem 1, Parts 1 and 2). Some work has to be done to make sure that these proofs still work for the allocation of heterogeneous objects; the additional proof steps that require *neutrality* in each part are key, and entirely new. Part 4 is very different from the corresponding proof part in Klaus and Nichifor (2020, Proof of Theorem 1, Part 4) due to the fact that we deal with heterogeneous instead of homogeneous objects. $\square$

The following examples present mechanisms that satisfy all the properties in Theorem 1, except for the one in the title of the example.

Let ${\widetilde{f}} \in {\mathcal {F}}$ be such that for all $i\in {\mathbb {N}}$, ${\widetilde{f}}_i=1$ (all reservation prices are equal to 1) and ${\widetilde{\vartriangleright }} \in {\mathcal {P}}$ be such that for any $i,j\in {\mathbb {N}}$, $i< j$ implies $i\mathbin {{\widetilde{\vartriangleright }}}j$ (lower indexed agents have higher priority). Most of our independence examples are modifications of the serial dictatorship mechanism with reservation prices based on ${\widetilde{f}}$ and ${\widetilde{\vartriangleright }}$, $\psi ^{({\widetilde{f}},{\widetilde{\vartriangleright }})}$.

### Example 1

(Minimal Tradability) The *no-trade mechanism* never allocates any real object and no payments are made.

### Example 2

(Individual Rationality) Mechanism ${\widetilde{\varphi }}$ is a variation of $\psi ^{({\widetilde{f}},{\widetilde{\vartriangleright }})}$ where only agent 1, if he is present, is treated differently in the allocation process that is based on $\psi ^{({\widetilde{f}},{\widetilde{\vartriangleright }})}$: agent 1 always pays his reservation price $f_1=1$, even if he is assigned the null object, and he only receives his best available real object $o\in O$ if $u_1(o,1)>u_1(0,1)$.

### Example 3

(Strategy-Proofness) Mechanism ${\widehat{\varphi }}$ is a variation of $\psi ^{({\widetilde{f}},{\widetilde{\vartriangleright }})}$ where ${\widehat{\alpha }}=\alpha ^{({\widetilde{f}},{\widetilde{\vartriangleright }})}$, but where agents who receive a real object $o\in O$ pay $\frac{1}{2}$ instead of 1.

### Example 4

(Consistency) Let $\vartriangleright \in {\mathcal {P}}$ such that $\vartriangleright \ne {\widetilde{\vartriangleright }}$. We apply $\psi ^{({\widetilde{f}},\vartriangleright )}$ to problems $\gamma \in \Gamma (N,O)$ where the set of agents *N* has cardinality 2, and $\psi ^{({\widetilde{f}},{\widetilde{\vartriangleright }})}$ otherwise.

### Example 5

(Independence of Unallocated Objects) We apply $\psi ^{({\widetilde{f}},{\widetilde{\vartriangleright }})}$ to each problem in which there are weakly less objects than agents who want them, and the no-trade mechanism (Example 1) otherwise.^10^

### Example 6

(Neutrality) Let $\vartriangleright ' \in {\mathcal {P}}$ be the priority orderings obtained from ${\widetilde{\vartriangleright }}$ by swapping the priorities of agents 1 and 2, i.e., at $\vartriangleright '$, agent 2 has the highest possible priority, and agent 1 has the highest possible priority after agent 2 (all other agents have lower priorities that do not change). We apply $\psi ^{({\widetilde{f}},{\widetilde{\vartriangleright }})}$ to problems in which agent 1 and object *o* are available, and object *o* is 1’s best real object $\mathrm {top}_1(O,{\widetilde{f}}_1)$, and $\psi ^{({\widetilde{f}},\vartriangleright ')}$ otherwise.^11^

### Example 7

(Non Wasteful Tie-Breaking) Let ${\overline{\psi }}^{({\widetilde{f}},{\widetilde{\vartriangleright }})}$ be a modification of $\psi ^{({\widetilde{f}},{\widetilde{\vartriangleright }})}$ such that an agent who is indifferent between [not receiving the null object and not paying anything] and [receiving his most preferred available real object and paying his reservation price] receives his most preferred available real object.

## Proof of Theorem 1

It is easy to see that any serial dictatorship mechanism with reservation prices, induced by some reservation price vector $f\in\cal{F}$ and some priority ordering $\succ\in\mathcal{P}$, satisfies all the properties in the theorem.

For the uniqueness proof we assume that $\varphi$ satisfies all the properties in the theorem; and, as announced in our proof sketch, we split the proof into four parts: first, we construct the individual reservation price vector $r\in {\mathcal {F}}$; second, we construct the priority ordering $\succ \in {\mathcal {P}}$ over ${\mathbb {N}}$; third, we prove that $\varphi =\psi ^{(r,\succ )}$ for single object problems; fourth, we extend the result that $\varphi =\psi ^{(r,\succ )}$ to any set of real objects $O\in {\mathcal {O}}$ via an induction argument.

### Part 1: individual reservation prices

For object allocation problems with one real object, Klaus and Nichifor (2020) have established the following lemma, which remains valid in our model.

#### Lemma 1

(Klaus and Nichifor 2020, Lemma 3) *Assume that mechanism* $\varphi$ *satisfies* *minimal tradability*, *individual rationality*, *and* *strategy-proofness*. *Consider a real object* $o\in {\mathbb {O}}$. *Then, for each agent* $i\in {\mathbb {N}}$, *there exists an individual reservation price* $r_{i,o}\ge 0$ *such that for each utility function* $u_i\in {\mathcal {U}}(\{i\},\{o\})$ *with associated valuation vector* $v_i\in {\mathcal {V}}(\{i\},\{o\})$:

(i) $$v_{i,o}> r_{i,o}$$ implies $$\varphi _i(\{i\},\{o\},u_i)=(o,r_{i,o})$$,
(ii) $v_{i,o}= r_{i,o}$ implies $\varphi _i(\{i\},\{o\},u_i)\in \{(0,0),(o,r_{i,o})\}$, and
(iii) $$v_{i,o}<r_{i,o}$$ implies $$\varphi _i(\{i\},\{o\},u_i)=(0,0)$$.

Next, we show that the individual reservation prices do not depend on which real object $o\in {\mathbb {O}}$ is used in the above lemma.

#### Lemma 2

*Assume that mechanism* $\varphi$ satisfies *minimal tradability*, *individual rationality*, *strategy-proofness*, and *neutrality*. *Then, for each agent* $i\in {\mathbb {N}}$ *and any two real objects* $o,{\hat{o}}\in {\mathbb {O}}$,

$$\begin{aligned} r_i:=r_{i,o}=r_{i,{\hat{o}}}, \end{aligned}$$

*where* $r_i$ *is agent* *i*’s *reservation price.*

#### Proof

Assume that mechanism $\varphi$ satisfies all the properties in the lemma. Let $i\in {\mathbb {N}}$ and consider $o,{\hat{o}}\in {\mathbb {O}}$. If $o={\hat{o}}$, then $r_{i,o}=r_{i,{\hat{o}}}$ follows trivially. Hence, assume that $o\ne {\hat{o}}$. Next, choose an (auxiliary) agent $j \in {\mathbb {N}}$, $i \ne j$. We will specify the allotments of both agents *i* and *j*, but note that only that of agent *i* matters for our proof.

Let $N=\{i,j\}$ and $O=\{o,{\hat{o}}\}$. By *minimal tradability*, there exist $u=(u_i,u_j) \in {\mathcal {U}}(N,O)$ with associated valuation vector $v = (v_i,v_j) \in {\mathcal {V}}(N,O)$ such that both real objects *o* and ${\hat{o}}$ are allocated. Without loss of generality, assume $\alpha _i(N,O,u)=o$ and $\alpha _j(N,O,u)={\hat{o}}$. Then, by *consistency* and Lemma 1 (i) and (ii),

$$\begin{aligned} \varphi _i(\{i,j\},\{o,{\hat{o}}\},u) =\varphi _i(\{i\},\{o\},u_{i,o}) =(o,r_{i,o}) \end{aligned}$$

and

$$\begin{aligned} \varphi _j(\{i,j\},\{o,{\hat{o}}\},u) =\varphi _j(\{j\},\{{\hat{o}}\},u_{j,{\hat{o}}}) =({\hat{o}},r_{j,{\hat{o}}}), \end{aligned}$$

respectively. In particular, note that

1 $$\begin{aligned} \pi _i(N,O,u)=r_{i,o} \text { and } \pi _j(N,O,u)=r_{j,{\hat{o}}}. \end{aligned}$$

Consider the relabelling $\sigma \in {\mathcal {S}}(O)$ such that $\sigma (o)={\hat{o}}$, $\sigma ({\hat{o}})=o$, and $\sigma (0)=0$; note that since there are only two real objects, $\sigma$ is the only possible relabelling. By *neutrality*,

$$\begin{aligned} \alpha _i(N,O,u^{\sigma })={\hat{o}}=\sigma (o)=\sigma (\alpha _i(N,O,u)) \end{aligned}$$

and

$$\begin{aligned} \alpha _j(N,O,u^{\sigma })=o= \sigma ({\hat{o}})=\sigma (\alpha _j(N,O,u)). \end{aligned}$$

Then, by *consistency* and Lemma 1 (i) and (ii),

$$\begin{aligned} \varphi _i(\{i,j\},\{o,{\hat{o}}\},u^{\sigma }) =\varphi _i(\{i\},\{{\hat{o}}\},u_{i,{\hat{o}}}^{\sigma }) =({\hat{o}},r_{i,{\hat{o}}}) \end{aligned}$$

and

$$\begin{aligned} \varphi _j(\{i,j\},\{o,{\hat{o}}\},u^{\sigma }) =\varphi _j(\{j\},\{o\},u_{j,o}^{\sigma }) =(o,r_{j,o}), \end{aligned}$$

respectively. In particular, note that

2 $$\begin{aligned} \pi _i(N,O,u^{\sigma })=r_{i,{\hat{o}}} \text { and } \pi _j(N,O,u^{\sigma })=r_{j,o}. \end{aligned}$$

By *neutrality*, we must also have

$$\begin{aligned} \pi _i(N,O,u^{\sigma })=\pi _i(N,O,u) \text{ and } \pi _j(N,O,u^{\sigma })=\pi _j(N,O,u), \end{aligned}$$

which by (1) and (2) implies that

$$\begin{aligned} r_{i,o}=r_{i,{\hat{o}}} \text{ and } r_{j,{\hat{o}}}=r_{j,o}. \end{aligned}$$

Since agent *j* and real objects *o* and ${\hat{o}}$ were arbitrarily chosen, it follows that agent *i* has a unique reservation price $r_i(=r_{i,o}=r_{i,{\hat{o}}})$. $\square$

By our next lemma, for any problem, if an agent receives a real object, then his valuation has to be weakly larger than his individual reservation price (which also equals his payment); otherwise, his payment is necessarily null.

#### Lemma 3

*Assume that mechanism* $\varphi$ *satisfies* *minimal tradability*, *individual rationality*, *strategy-proofness*, *consistency*, *independence of unallocated objects*, *and* *neutrality*. *Then, for each* $(N,O) \in {\mathcal {N}}\times {\mathcal {O}}$, *each* $\gamma \in \Gamma (N,O)$ *with associated valuation vector* $v\in {\mathcal {V}}(N,O)$, *each* $i\in N$, *and each* $o\in O$, *if* $\alpha _i(\gamma )=o$, *then* $\pi _i(\gamma )=r_i\le v_{i,o}$ *(with* $r_i$ *as obtained in Lemma* 2).

#### Proof

Assume that mechanism $\varphi$ satisfies all the properties in the lemma. Let $(N,O) \in {\mathcal {N}}\times {\mathcal {O}}$, and $\gamma =(N,O,u)\in \Gamma (N,O)$ with associated valuation vector $v\in {\mathcal {V}}(N,O)$. Let $i\in N$, $o\in O$, and $\alpha _i(\gamma )=o$. If all agents but agent *i* leave with their allotments, then the reduced problem is $\gamma _{\{i\}}=(\{i\},O_{\{i\}},u_i)$ where $O_{\{i\}}=O{\setminus }\bigcup _{j\in N{\setminus }\{i\}}\alpha _j(\gamma )$. By *consistency*, $\varphi _i(\gamma _{\{i\}})=\varphi _i(\gamma )$ and $\alpha _i(\gamma _{\{i\}})=\alpha _i(\gamma )=o$. If $O_{\{i\}}=\{o\}$, then $\gamma _{\{i\}}=(\{i\},\{o\},u_i)$. If $\{o\}\varsubsetneq O_{\{i\}}$, then using *independence of unallocated objects*, we obtain $\varphi _i(\gamma _{\{i\}})=\varphi _i(\{i\},\{o\},u_i)$.

Thus, $\varphi _i(\{i\},\{o\},u_i)=\varphi _i(\gamma )$ and $\alpha _i(\{i\},\{o\},u_i)=\alpha _i(\gamma )=o$. By Lemma 1, $v_{i,o}\ge r_i$ and $\varphi _i(\{i\},\{o\},u_i)=(o,r_i)=\varphi _i(\gamma )$. In particular, $\pi _i(\gamma )=r_i\le v_{i,o}$. $\square$

### Part 2: priority ordering

For object allocation problems with one real object, Klaus and Nichifor (2020, Proof of Theorem 1, Part 2) have shown that for any $o \in {\mathbb {O}}$, there exists a (transitive) priority ordering $\succ _o \in {\mathcal {P}}$ over ${\mathbb {N}}$. More specifically, for any two agents $i,j\in {\mathbb {N}}$ and any real object $o \in {\mathbb {O}}$, let $N=\{i,j\}$ and fix a utility profile $({\bar{u}}_i,{\bar{u}}_j)\in {\mathcal {U}}(N,\{o\})$ such that the associated valuation vector is ${\bar{v}}=(r_i+1,r_j+1)$. Then,

3 $$\begin{aligned} i\succ _o j \text{ if } \text{ and } \text{ only } \text{ if } \alpha _i(N,\{o\},({\bar{u}}_i,{\bar{u}}_j))=o, \end{aligned}$$

a result that remains valid in our model.

Next, we show that the priority ordering over agents does not depend on which real object $o\in {\mathbb {O}}$ is considered, i.e., we show that for two distinct real objects $o,{\hat{o}} \in {\mathbb {O}}$,

$$\begin{aligned} \succ _o=\succ _{{\hat{o}}}. \end{aligned}$$

Specifically, we show that for any two agents $i,j \in {\mathbb {N}}$, $i \neq j$, we have $i\succ_o j\mbox{ if \;and\; only\; if }i\succ_{\hat{o}} j.$

Let $N=\{i,j\}$ and consider the problem where both objects are available, i.e., $O=\{o,{\hat{o}}\}$. Assume further that both agents would like to have object *o* while they are not interested in object ${\hat{o}}$, i.e., the utility profile $u\in {\mathcal {U}}(N,O)$ is such that $u=(u_i,u_j)=\left( ({\bar{u}}_{i,o},u^0_{i,{\hat{o}}}),({\bar{u}}_{j,o},u^0_{j,{\hat{o}}})\right)$ with associated valuation vector $v=((r_i+1,0),(r_j+1,0)) \in {\mathcal {V}}(N,O)$. We consider problem (*N*, *O*, *u*).

First, we show that object ${\hat{o}}$ is not allocated. Suppose, by contradiction, that for $x\in N$, $\alpha _x(N,O,u)={\hat{o}}$. Since $v_{x,{\hat{o}}}=0$ and prices are non-negative, by *individual-rationality* (IR2), $\pi _x(N,O,u)=0$. By *non-wasteful tie-breaking*, $\alpha _x(N,O,u)=0$ (otherwise, if $\alpha _x(N,O,u)={\hat{o}}$, we would have $u_x({\hat{o}},\pi _x(N,O,u))=u_x(0,0)$; a contradiction). Hence, for both agents $x\in N$, $\alpha _x(N,O,u)\ne {\hat{o}}$.

Second, we show that object *o* has to be allocated to agent *i*. When removing unallocated object ${\hat{o}}$ from problem (*N*, *O*, *u*) we obtain problem $(N,\{o\},u_{\{o\}})$; by *independence of unallocated objects*, we have that $\alpha _i(N,O,u)=\alpha _i(N,\{o\},u_{\{o\}})$. Since problem $(N,\{o\},u_{\{o\}})=(N,\{o\},({\bar{u}}_i,{\bar{u}}_j))$, by (3), $i \succ _o j$ implies $\alpha _i(N,O,u)=o$.

Third, consider the relabelling $$\sigma \in {\mathcal {S}}(O)$$ such that $$\sigma (o)={\hat{o}}$$, $$\sigma ({\hat{o}})=o$$, and $$\sigma (0)=0$$; note that since there are only two real objects, $$\sigma $$ is the only possible relabelling. The relabelling of the utility profile is $$u^\sigma=(u^\sigma_i,u^\sigma_j) =\left((u^0_{i,\hat{o}},\bar{u}_{i,o}),(u^0_{j,\hat{o}},\bar{u}_{j,o})\right)\in
\mathcal{U}(N,O)$$ with associated valuation vector. $$\left( (0,r_i+1),(0,r_j+1)\right) \in {\mathcal {V}}(N,O)$$. By *neutrality*,

$$\begin{aligned} \alpha _i(N,O,u^{\sigma })={\hat{o}}=\sigma (o)=\sigma (\alpha _i(N,O,u)) \end{aligned}$$

and

$$\begin{aligned} \alpha _j(N,O,u^{\sigma })=0=\sigma (0)=\sigma (\alpha _j(N,O,u)), \end{aligned}$$

which by (3) implies $i \succ _{{\hat{o}}} j$.

Thus, for any distinct agents $i,j \in {\mathbb {N}}$ and any distinct real objects $o,{\hat{o}} \in {\mathbb {O}}$, if $i \succ _o j$, then $i \succ _{{\hat{o}}} j$, which implies that $\succ _o=\succ _{{\hat{o}}}$.

We denote the unique priority ordering over agents by $\succ$.

### Part 3: single-object problems

For object allocation problems with one real object, Klaus and Nichifor (2020, Proof of Theorem 1, Part 3) have shown that $\varphi$ always assigns the object and payments as if it is a serial dictatorship mechanism based on $r\in {\mathcal {F}}$ (from Part 1) and $\succ \in {\mathcal {P}}$ (from Part 2); their result remains valid in our model.

### Part 4: an arbitrary set of real objects

We now show by induction on the number of objects that $\varphi =\psi ^{(r,\succ )}$ for the general domain of all problems.

**Induction basis** $\mathbf{|O|=0,1}$: Let $N \in {\mathcal {N}}$, $O\in {\mathcal {O}}$, and $\gamma =(N,O,u) \in \Gamma (N,O)$ such that $|O|=0,1$. Then, $\varphi (\gamma )=\psi ^{(r,\succ )}(\gamma )$ follows for $|O|=0$ by *individual rationality* (IR1) and for $|O|=1$ by Part 3.

**Induction hypothesis** $\mathbf{|O|\le k}$: On the subdomain of problems where at most $k\ge 1$ real objects are available, we assume $\varphi =\psi ^{(r,\succ )}$.

**Induction step** $\mathbf{|O|=k+1}$: We show that for problems where $k+1$ real objects are available, we have $\varphi =\psi ^{(r,\succ )}$. Let $\varphi =(\alpha ,\pi )$ and $\psi ^{(r,\succ )}=(\alpha ',\pi ')$.

Consider a set of agents $N\in {\mathcal {N}}$, a set of real objects $O\in {\mathcal {O}}$ such that $|O|=k+1$, and a utility profile $u\in {\mathcal {U}}(N,O)$ with associated valuation vector $v\in {\mathcal {V}}(N,O)$. If no agent in *N* would like to receive a real object at problem (*N*, *O*, *u*), i.e., if for all $i\in N$ and $o\in O$, $v_{i,o}\le r_i$, then by Lemma 3 and *non-wasteful tie-breaking*, for all $i\in N$, $\varphi_i(N,O,u)=(0,0)=\psi_i^{(r,\succ)}(N,O,u)$. Hence, assume that for some agent $i\in N$ and some real object $o\in O$, $v_{i,o}> r_i$. Without loss of generality assume that agent 1 is the highest priority agent in *N* according to $\succ$ such that for some real object $o\in O$, $v_{1,o}> r_1$.

First, we show that agent 1’s allotment under both mechanisms $\varphi$ and $\psi ^{(r,\succ )}$ has to be the same; then, we show that the allotment of all other $N {\setminus } \{1\}$ agents under both mechanisms $\varphi$ and $\psi ^{(r,\succ )}$ is also the same.

*Claim 1:* $$\varphi _1(N,O,u)=\psi ^{(r,\succ )}_1(N,O,u)$$.

We start by showing that $\alpha _1(N,O,u)=\alpha _1'(N,O,u)$. Let ${\hat{o}}:=\mathrm {top}_1(O,r_1)$. Thus, $\psi _1^{(r,\succ )}(N,O,u)=({\hat{o}},r_1)$ and in particular, $\alpha '_1(N,O,u)={\hat{o}}$.

Assume, by contradiction, that $\alpha _1(N,O,u)\ne {\hat{o}}$.

Consider a utility function $u'_1=\left( u_{1,{\hat{o}}},(u^{-\infty }_{1,o'})_{o'\in O{\setminus }\{{\hat{o}}\}}\right) \in {\mathcal {U}}(\{1\},O)$ with valuation vector $v'_1=(v_{1,{\hat{o}}},-\infty ,\ldots ,-\infty )$, i.e., $v'_{1,{\hat{o}}}=v_{1,{\hat{o}}}$ and for all $o' \in O{\setminus }\{{\hat{o}}\}$, $v'_{1,o'}=-\infty$. Let $u'=(u'_1,u_{-1})$. Then, by *strategy-proofness*, $\alpha _1(N,O,u')\ne {\hat{o}}$ and $\alpha '_1(N,O,u')={\hat{o}}$. Additionally, using Lemma 3, $\alpha _1(N,O,u')=0$.

*Case 1.* There exists an agent $i\in N{\setminus }\{1\}$ such that $\alpha _i(N,O,u')={\hat{o}}$.

Recall that $\alpha _1(N,O,u')=0$. Hence, when all agents except agents 1 and *i* leave with their allotments, we obtain the reduced problem $(\{1,i\},O',u'_{\{1,i\}})$ where ${\hat{o}}\in O'\subseteq O$. By *consistency*, we then have

$$\begin{aligned} \alpha _1(N,O,u')=\alpha _1(\{1,i\},O',u'_{\{1,i\}}). \end{aligned}$$

Note that only object ${\hat{o}}$ is allocated. Hence, when removing all unallocated objects from reduced problem $(\{1,i\},O',u'_{\{1,i\}})$, we obtain the problem $(\{1,i\},\{{\hat{o}}\},u'_{\{1,i\}})$. By *independence of unallocated objects*, we then have

$$\begin{aligned} \alpha _1(\{1,i\},O',u'_{\{1,i\}})=\alpha _1(\{1,i\},\{{\hat{o}}\},u'_{\{1,i\}}). \end{aligned}$$

In particular it follows that

$$\begin{aligned} \alpha _1(\{1,i\},\{{\hat{o}}\},u'_{\{1,i\}})=\alpha _1(N,O,u')=0, \end{aligned}$$

contradicting the Induction Basis (since for problems with one real object, agent 1 as the highest priority agent who wants the object should receive it).

*Case 2.* For all agents $i\in N{\setminus }\{1\}$, $\alpha _i(N,O,u')\ne {\hat{o}}$.

Recall that $\alpha _1(N,O,u')=0$. Hence, when all agents except agent 1 leave with their allotments, we obtain the reduced problem $(\{1\},O'',u'_{1})$ where ${\hat{o}}\in O''\subseteq O$. By *consistency*, we then have

$$\begin{aligned} \alpha _1(N,O,u')=\alpha _1(\{1\},O'',u'_{1}). \end{aligned}$$

We now remove the set of unallocated real objects $O''{\setminus }\{{\hat{o}}\}$ and obtain the problem $(\{1\},\{{\hat{o}}\},u'_{1})$. By *independence of unallocated objects*, we then have

$$\begin{aligned} \alpha _1(\{1\},O'',u'_{1})=\alpha _1(\{1\},\{{\hat{o}}\},u'_{1}). \end{aligned}$$

In particular, it follows that

$$\begin{aligned} \alpha _1(\{1\},\{{\hat{o}}\},u'_{1})=\alpha _1(N,O,u')=0, \end{aligned}$$

contradicting the Induction Basis (since for problems with one real object, agent 1 as the highest priority agent who wants the object should receive it).

*Cases 1* and *2* now imply that $\alpha _1(N,O,u')={\hat{o}}=\alpha _1(N,O,u)$. Thus, as $\alpha '_1(N,O,u)={\hat{o}}$, we have now shown that $\alpha _1(N,O,u)=\alpha '_1(N,O,u)$. By Lemma 3, $\varphi _1(N,O,u)=\psi ^{(r,\succ )}_1(N,O,u)=({\hat{o}},r_1)$, completing the proof of our claim.

*Claim 2:* for each $i \in N {\setminus } \{1\}, \varphi _i(N{\setminus }\{1\},O_{N{\setminus }\{1\}},u_{N{\setminus }\{1\}})=\psi ^{(r,\succ )}_i(N{\setminus }\{1\},O_{N{\setminus }\{1\}},u_{N{\setminus }\{1\}})$.

When agent 1 leaves problem (*N*, *O*, *u*) with his allotment under both mechanisms $\varphi$ and $\psi ^{(r,\succ )}$, we obtain the reduced problem $(N{\setminus }\{1\},O_{N{\setminus }\{1\}},u_{N{\setminus }\{1\}})$ . By *consistency*, for all $i\in N{\setminus }\{1\}$, we then have

$$\begin{aligned} \varphi _i(N,O,u) =\varphi _i(N{\setminus }\{1\},O_{N{\setminus }\{1\}},u_{N{\setminus }\{1\}}) \end{aligned}$$

and

$$\begin{aligned} \psi ^{(r,\succ )}_i(N,O,u) =\psi ^{(r,\succ )}_i(N{\setminus }\{1\},O_{N{\setminus }\{1\}},u_{N{\setminus }\{1\}}). \end{aligned}$$

By the Induction Hypothesis, for all $i\in N{\setminus }\{1\}$, we have

$$\begin{aligned} \varphi _i(N{\setminus }\{1\},O_{N{\setminus }\{1\}},u_{N{\setminus }\{1\}}) =\psi ^{(r,\succ )}_i(N{\setminus }\{1\},O_{N{\setminus }\{1\}},u_{N{\setminus }\{1\}}). \end{aligned}$$

Taken together, our claims yield the desired result, i.e., $\varphi (N,O,u)=\psi ^{(r,\succ )}(N,O,u)$. $\square$

## Footnotes

1 In Sect. 4, we discuss in detail the technical similarities and the differences between our Theorem 1 and the main result of Klaus and Nichifor (2020, Theorem 1), as well as the role played by *neutrality*.

2 We more precisely place each of the properties that we use within the relevant literature in Sect. 2, immediately after we formally introduce each property.

3 Finite sets of potential agents and potential real objects would not change any of our results.

4 Requiring continuity of $u_i$ would be a less general assumption that guarantees the existence of valuation vector $v_i$.

5 Klaus and Nichifor (2020) define *minimal tradability* by requiring that (i) if there are at least as many agents as objects, then there is some utility profile for which all objects are allocated and (ii) if there are more objects than agents, then there is some utility profile at which each agent receives an object. Our definition of *minimal tradability* is in character strictly weaker than that of Klaus and Nichifor (2020) because we drop their second requirement.

6 Strictly speaking, the notation $u_{N{\setminus } M}$ has only been introduced for the removal of agents *M* from utility profile *u* but in the context of *consistency*, the additional adjustment to a smaller set of objects should neither be confusing nor require additional notation.

7 Note that if the reservation prices are zero for all agents, we obtain the classical serial dictatorship mechanism. That is, given the reservation price vector $\varvec{0} = (0,0,\ldots )\in {\mathcal {F}}$ and a priority ordering $\vartriangleright \in {\mathcal {P}}$, $\psi ^{(\varvec{0},\vartriangleright )}$ is a *serial dictatorship mechanism*.

8 For instance, two mole removal surgeries that are otherwise identical except for the fact that one is scheduled for next Monday, and the other one for next Tuesday, would yield two heterogeneous objects.

9 However, for genuinely different objects (e.g., a mole removal surgery versus a mastectomy), *neutrality* would be a very strong requirement.

10 We say that an agent wants an object if his valuation is higher than his reservation price for it. The cases (i) weakly less objects than agents who want them, and (ii) more objects than agents who want them, are mutually exclusive and exhaustive. Note that removing agents together with their allotments cannot switch a problem between cases (i) and (ii); thus, *consistency* is satisfied. Meanwhile, removing unallocated objects may switch a problem from (ii) to (i); thus, *independence of unallocated objects* is violated.

11 To see that *strategy-proofness* is satisfied, note that the different priorities ${\widetilde{\vartriangleright }}$ and $\vartriangleright '$ only matter when agents 1 and 2 have the same best (acceptable) object: if that best object is *o*, then agent 1 receives it and he has no incentive to misrepresent his preferences; otherwise, if that best object is different from *o*, then agent 1 receives his second best acceptable object or the null object (and again, he has no incentive to misrepresent his preferences). The case of agents 1 and 2 having the same best (acceptable) object, object *o* versus an object $o'\ne o$, also illustrates why *neutrality* is violated.
